# Course of uncomplicated acute gastroenteritis in children presenting to out-of-hours primary care

**DOI:** 10.1186/s12875-022-01739-2

**Published:** 2022-05-24

**Authors:** Anouk A. H. Weghorst, Irma J. Bonvanie, Gea A. Holtman, Michiel R. de Boer, Marjolein Y. Berger

**Affiliations:** 1 Department of General Practice and Elderly Care Medicine, FA21, University of Groningen, University Medical Centre Groningen, PO Box 196, 9700 AD Groningen, The Netherlands; 2 Department of Paediatrics, University of Groningen, University Medical Centre Groningen, Groningen, The Netherlands

**Keywords:** Safety netting, Symptoms, Vomiting, Diarrhea, Fever

## Abstract

**Background:**

The aim of this article is to describe the courses of vomiting, diarrhea, fever, and clinical deterioration, in children with uncomplicated gastroenteritis at presentation. This study was performed as a 7-day prospective follow-up study in an out-of-hours primary care service. The course of vomiting, diarrhea, and fever was analyzed by generalized linear mixed modeling. Because young children (≤ 12 months) and children with severe vomiting are at increased risk of dehydration, the potentially more complicated courses of these groups are described separately. The day(s) most frequently associated with deterioration and the symptoms present in children who deteriorated during follow-up were also described.

**Results:**

In total, 359 children presented with uncomplicated acute gastroenteritis to the out-of-hours primary care service. Of these, 31 (8.6%) developed a complicated illness and needed referral or hospitalization. All symptoms decreased within 5 days in most children (> 90%). Vomiting and fever decreased rapidly, but diarrhea decreased at a somewhat slower pace, especially among children aged 6–12 months. Children who deteriorated during follow-up had a higher frequency of vomiting at presentation and higher frequencies of vomiting and fever during follow-up.

**Conclusions:**

The frequency of vomiting, not its duration, appears to be the more important predictor of deterioration. When advising parents, it is important to explain the typical symptom duration and to focus on alarm symptoms. Clinicians should be vigilant for children with higher vomiting frequencies at presentation and during follow-up because these children are more likely to deteriorate.

**Supplementary Information:**

The online version contains supplementary material available at 10.1186/s12875-022-01739-2.

## What is already known on this topic

- No clear safety net can be communicated to parents of children with acute gastroenteritis, because little is known about the expected duration of symptoms in an uncomplicated course of the disease

## What this study adds

- This study reveals that vomiting, diarrhoea and fever should be over in 5 days after presentation, except for children of 6–12 months old in whom diarrhoea should be over in 7 days after presentation
- Children who deteriorated during follow-up had higher frequencies of vomiting at presentation and had higher frequencies of vomiting and fever during follow-up.

## Introduction

Acute gastroenteritis is a common childhood disease that contributes significantly to the burden of primary care consultations [1–3]. Characterized by vomiting and/or diarrhea with or without fever [4, 5], it typically results in an uncomplicated minor illness that can be managed safely at home [3, 6]. However, it can also lead to severe dehydration, particularly in young children and in children with severe vomiting [5, 7]. Given these risks, safety netting is recommended for children with acute gastroenteritis who do not require referral [8].

Safety netting advice should include clear parental education about the expected disease course, possible alarm symptoms, and when and where to seek further help [9]. The goal of safety netting is to increase parental self-efficacy to take care of their ill child while ensuring that children who deteriorate are re-evaluated [10]. Ideally, advice should be tailored to each child, taking into account risk factors for dehydration and a more complicated illness course, such as young age (≤ 12 months) and severe vomiting [5, 8]. There is evidence that safety netting reduces the reattendance of febrile children in primary care [10]. However, a lack of knowledge about the expected duration of symptoms in an uncomplicated disease course means that current advice is not comprehensive. It is also unclear when deterioration occurs, and indeed, what symptoms are typically present at that time. Improving the knowledge of the expected course of acute gastroenteritis could help both general practitioners (GPs) and parents to distinguish children in need of re-evaluation or referral from among the vast number who will have an uncomplicated course.

In this study, we aimed to describe the courses of vomiting, diarrhea, fever, and clinical deterioration in children for 7 days after presenting to primary care with uncomplicated gastroenteritis.

## Methods

### Design and setting

This study used data obtained for a previous cohort study and a randomized controlled trial (RCT) for evaluating the (cost-)effectiveness of oral ondansetron added to care-as-usual [11, 12]. The original research was conducted at three out-of-hours primary care (OOH-PC) centers in the north of the Netherlands from 2015 to 2018. A detailed description of the study design has been described elsewhere [13]. All parents of the included children gave written informed consent. The Medical Ethics Review Committee of the University Medical Centre of Groningen approved this study (NL5830).

### Participants

Children were included in the RCT if they were aged 6 months to 6 years, had a diagnosis of acute gastroenteritis, and were considered at risk of dehydration [5], which was based on two criteria: 1) ≥ 4 vomiting episodes in the 24 h before attending the OOH-PC center; and 2) ≥ 1 vomiting episode in the 4 h before attending the OOH-PC center. Antiemetic use or prescription in the 6 h before presentation was the main exclusion criteria for the RCT. Included children were randomly allocated to either care-as-usual (oral rehydration therapy) or care-as-usual plus one dose of 0.1 mg/kg oral ondansetron [11, 12]. The only inclusion criteria for the parallel cohort were that the child was age 6 months to 6 years and had a diagnosis of acute gastroenteritis. All parents of children from the cohort study and RCT completed a diary for 7 days.

Data of children included in the RCT and cohort were included in the current study if the children had uncomplicated acute gastroenteritis at presentation. A complicated illness was defined as requiring referral to, or hospitalization in, a pediatric emergency department immediately after presentation. Children referred at baseline were therefore excluded.

### Patient recruitment and baseline assessment

Parents of consecutive children presenting to the OOH-PC with vomiting and/or diarrhea were informed about the studies by a research assistant before the GP consultation. If parents were interested, the research assistant started baseline assessment and collected demographic and medical data. Subsequently, the GP confirmed or refuted the diagnosis of acute gastroenteritis and assessed the degree of dehydration. Children were included by the research assistant based on the GP’s diagnosis, the baseline data, and receipt of informed consent from parents.

### Outcomes

The primary outcome was to describe the courses of vomiting, diarrhea, and fever over the 7-day follow-up period among children with uncomplicated acute gastroenteritis. Secondary outcomes were the day on which deterioration occurred and the prevalence of each symptom on the day of deterioration.

### Measurements

Parents were asked to complete a diary for 7 days. In the first 4 h, they were asked to report on their child’s progress and any health care use each hour; thereafter, they reported on these daily until 7 days after presentation. Data from the first day of the diary were omitted from analysis because they only accounted for the first 4 h and not a full 24-h period, as reported for all other days.

In the diary, parents state if each symptom had been present in the past 24 h (yes/no). A vomiting episode was defined as the forceful expulsion of stomach contents [14]. Diarrhea was defined as the passage of three or more loose or liquid stools per day (Bristol type 6 or 7) [5, 8]. Fever was defined as a body temperature of 38.0 °C or more. Because young children (≤ 12 months) and those with severe vomiting are at increased risk of dehydration, and thereby a complicated course, the courses for these groups were described separately [5, 8]. Deterioration was defined as referral or admission to hospital during follow-up. We recorded the day of deterioration and the symptoms present on the follow-up days.

### Statistical analysis

Descriptive statistics were used to report the baseline characteristics, including the risk factors and alarm symptoms of dehydration. Baseline data are reported as medians and interquartile ranges (IQR) or as numbers and percentages.

The courses of vomiting, diarrhea, and fever were analyzed by generalized linear mixed models (GLMMs). First, we created a new variable with child subgroups from a 2 × 2 cross-tabulation of age (≤ 12 months versus > 12 months) and severe vomiting (yes versus no). This new variable, time (in days), the interaction between these variables, and ondansetron use (yes versus no), were set as fixed effects. Ondansetron use was included to adjust for potential confounding by medication use. As ondansetron was associated with an increase in episodes of diarrhea, we additionally checked this for our population [15]. We accounted for repeated measures by including a random intercept at the child level, and we assumed missing data to be missing at random. Estimated percentages and 95% confidence intervals are presented for the GLMM.

Frequency of deterioration is described by day of follow-up, using bar charts, and we describe the differences in baseline characteristics between children who did and did not deteriorate during follow-up. In addition, the presence of vomiting, diarrhea, and fever were compared between groups.

Data were analyzed using IBM SPSS, version 25.0 (IBM Corp., Armonk, NY, US).

## Results

### Participant flow and baseline characteristics

**Fig. 1:**
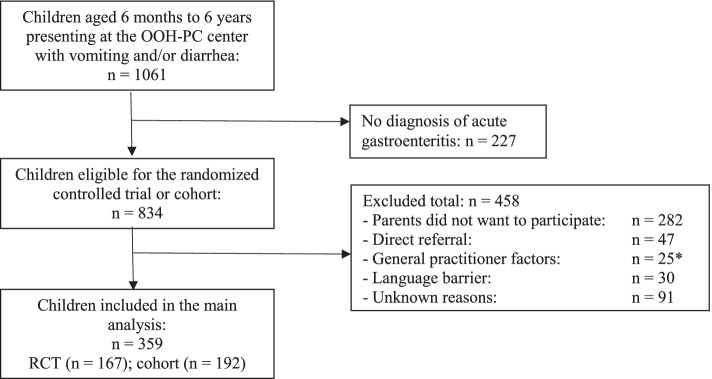
Flow of participants. *GP objected to ondansetron use (*n* = 16) or did not agree with inclusion (*n* = 9). Abbreviations: GP, general practitioner; OOH-PC, out-of-hours primary care; RCT, randomized controlled trial

Participant flow is shown in Fig. 1. In total, 1061 children were screened for eligibility at one of the three participating OOH-PC centers. Finally, data for 359 children were used in the analyses. Their median age was 1.5 years (IQR, 0.9–2.2 years) and 184 (51.3%) were female.

The median duration of vomiting before presentation was 2 days (IQR, 1.0–3.0 days). Diarrhea was present in 181 (50.7%) children and the median duration before presentation was 3 days (IQR, 2.0–4.0 days) (Table 1). Severe vomiting and age 6–12 months were the most common risk factors for dehydration, being present in 244 (68.0%) and 103 (28.7%) children, respectively. The most frequent alarm symptom for dehydration was no urine output for 24 h, which was present in 45 (13.3%) children (Table 2).

**Table 1:** Baseline characteristics

**Baseline characteristics**	N	**Included**	N	**Deterioration follow-up**	N	**Hospitalized follow-up**
Gender (female)	359	184 (51.3)	31	15 (48.4)	18	8 (44.4)
Age in years	359	1.5 (0.9–2.2)	31	1.6 (0.8–2.5)	18	1.5 (0.8–2.0)
Weight in kg	296	11.1 (9.5–14.0)	29	11.5 (9.5–13.6)	17	10.0 (9.5–12.8)
Vomiting present	357	328 (91.9)	31	29 (93.5)	18	17 (94.4)
Duration of vomiting prior to presentation OOH-PC in days	326	2.0 (1.0–3.0)	29	2.0 (1.0–3.0)	17	1.0 (0.9–2.5)
Frequency of vomiting past 24 h	311	5.0 (3.0–8.0)	29	6.0 (3.0–17.0)	17	9.0 (3.5–18.0)
Diarrhea present	357	181 (50.7)	31	14 (45.2)	18	10 (55.6)
Duration of diarrhea prior to presentation OOH-PC in days^a^	180	3.0 (2.0–4.0)	13	2.0 (1.5–3.0)	9	3.0 (1.5–5.0)
Frequency of diarrhea in past 24 hours^a^	167	5.0 (4.0–7.0)	14	5.0 (3.0–8.5)	10	5.0 (3.0–8.5)
Dehydration assessed by GP (0–100%)	339	20.0 (9.0–35.0)	31	20.0 (10.0–45.0)	18	20.0 (7.8–54.5)
Additional risk factors for dehydration^b^	357		31		18	
1		131 (36.7)		13 (41.9)		7 (38.9)
≥ 2		30 (8.4)		3 (9.6)		2 (11.1)
Alarm symptoms of dehydration^c^	357		31		18	
1		50 (14.0)		8 (25.8)		6 (33.3)
≥ 2		8 (2.2)		1 (3.2)		1 (5.6)

Results are shown as Median (IQR) or N (%)

*Abbreviations*: *OOH-PC* Out-of-hours primary care, *GP* General practitioner, *IQR* interquartile range

^a^Numbers are only presented for participants with diarrhea

^b^Risk factors assessed at baseline: ≥ 6 watery stools or diarrhea, fever, and reduced intake

^c^Alarm symptoms assessed at baseline: confusion or decreased consciousness, bradycardia, weak peripheral heartbeat pulsations, capillary refill > 4 s, skin pitch > 4 s, extremities cold/marbled, and no urine output for 24 h

**Table 2:** Risk factors and alarm symptoms of dehydration

	N	**Included**	N	**Deterioration follow-up**	N	**Hospitalized follow-up**
**Risk factors for dehydration**						
Age 6–12 months	359	103 (28.7)	31	8 (25.8)	18	5 (27.8)
Severe vomiting^a^	359	244 (68.0)	31	25 (80.6)	18	15 (83.3)
≥ 6 watery stools	355	81 (22.8)	31	6 (19.4)	18	5 (27.8)
Fever (≥ 38 °C)	346	84 (24.3)	31	11 (35.5)	18	4 (22.2)
Reduced intake in the last 12 h	353	28 (7.9)	31	3 (9.7)	18	3 (16.7)
**Alarm symptoms of dehydration**						
Confused or decreased consciousness	357	10 (2.8)	31	2 (6.5)	18	2 (11.1)
Bradycardia	354	1 (0.3)	31	0 (0.0)	18	(0.0)
Weak peripheral pulse	353	0 (0.0)	31	0 (0.0)	18	(0.0)
Capillary refill > 4 s	356	1 (0.3)	31	1 (3.2)	18	1 (5.6)
Skin pitch > 4 s	356	1 (0.3)	31	1 (3.2)	18	1 (5.6)
Extremities cold/marbled	356	7 (2.0)	31	0 (0.0)	18	(0.0)
No urine output for 24 h	338	45 (13.3)	29	6 (20.7)	16	4 (25.0)

Results are shown as N (%)

^a^Severe vomiting is defined as at least four episodes of vomiting in the 24 h before presentation and at least one episode of vomiting in the 4 h before presentation

### Presence of symptoms

**Fig. 2:**
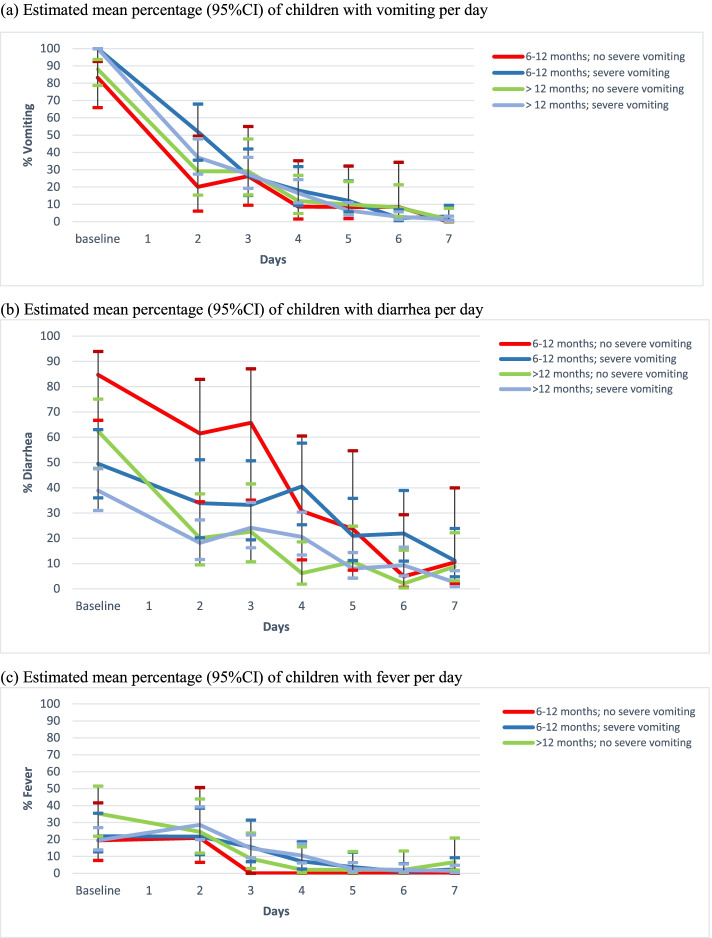
Estimated percentages of children with vomiting, diarrhea, and fever over time **a** Estimated mean percentage (95%CI) of children with vomiting per day. Abbreviations: 95%CI, 95% confidence interval. **b** Estimated mean percentage (95%CI) of children with diarrhea per day. **c** Estimated mean percentage (95%CI) of children with fever per day

Grouping children by age and vomiting severity produced four groups: age 6–12 months without severe vomiting (*n* = 32), age 6–12 months with severe vomiting (*n* = 71), age > 12 months without severe vomiting (*n* = 83), and age > 12 months with severe vomiting (*n* = 173). Estimated percentages and 95% confidence intervals for vomiting, diarrhea, and fever are presented in Fig. 2 and Additional file 1.

Most children presented with vomiting, and 20%–50% of these were still vomiting by day 2 after presentation, with the highest percentages among children with severe vomiting at presentation. By day 5, irrespective of risk group, these percentages had decreased to 10% (Fig. 2a).

The percentage of children with diarrhea at presentation varied by age and the presence of severe vomiting. The lowest percentage was 38.9% for children aged > 12 months with severe vomiting and the highest was 84.7% for children aged 6–12 months without severe vomiting. Notably, 10% of children aged > 12 months had persistent diarrhea by day 5, but this threshold was only reached by day 7 for children aged 6–12 months (Fig. 2b). There was no association found between ondansetron use and an increase in diarrhea episodes.

Fever was present in 20%–40% of children at presentation, with < 10% having persistent fever at day 4. The course of fever was broadly comparable in all groups (Fig. 2c).

### Deterioration: referral or hospitalization

During follow-up, 31 children (8.6%) were referred to the emergency department and 18 (5.0%) of these were hospitalized. Most children deteriorated on days 2 and 3 after presenting (Additional file 2). Children who were hospitalized had a median of 1 day of vomiting prior to presentation compared to 2 days in children who were not hospitalized during follow-up; however, hospitalized children had higher median frequencies of vomiting at presentation (9 vs 5 in 24 h) (Table 1). During follow-up, children who deteriorated had higher frequencies of vomiting and fever, but the frequencies of diarrhea throughout follow-up were similar to those of children who did not deteriorate (Additional file 3).

## Discussion

### Summary

This study described the courses of vomiting, diarrhea, and fever over 7 days, together with the pattern of clinical deterioration, among children who present to OOH-PC centers with uncomplicated acute gastroenteritis. In total, 8.6% of children developed a complicated illness that required referral and 5.0% were hospitalized. Symptoms decreased by day 5 in > 90% of the children, except for diarrhea in children aged 6–12 months. Vomiting and fever decreased rapidly while diarrhea decreased at a slower pace, especially among younger children. There was a higher frequency of vomiting at presentation, and the symptoms of vomiting and fever persisted for longer, among children who deteriorated.

### Limitations and strengths

A limitation of this is study is that the RCT focused on children who presented with excessive vomiting, indicating that children with severe vomiting could have been overrepresented, which in turn, could have influenced the observed course of the illness and its deterioration. However, we formed several analysis subgroups and separately evaluated the illness courses of children with and without severe vomiting. This design has the added benefit of enabling us to give advice tailored to a child’s specific situation.

Despite this limitation, the study benefited from using prospectively collected data on daily progress and healthcare use for 359 children who presented with an uncomplicated course at the OOH-PC. The use of a parental diary over 7 days enabled us to gain insight into the courses of vomiting, diarrhea, and fever among children with uncomplicated illnesses at presentation.

### Comparison with existing literature

In this study, we tried to provide good safety netting advice for children with acute gastroenteritis in primary care. Thompson et al. already found that no diagnostic test or clinical decision rule in general practice is 100% sensitive [9]. The course of diseases differs between individuals and safety netting is therefore extremely important to give a diagnostic strategy to deal with diseases in primary care.

Over 90% of children stopped vomiting within 5 days after presentation in this study. Chow et al. reported that vomiting persisted for a mean duration of 1.84 days, which is far shorter than in our study. One reason for this discrepancy might be the difference in etiology, with rotavirus known to cause illness that typically persists for 5 to 7 days [16]. Leung et al. reported that vomiting was almost four times more common and tended to be prolonged among children with rotavirus gastroenteritis compared with other etiological agents [16, 17]. Given that an inclusion criterion for our RCT was severe vomiting, it is possible that more children with rotavirus gastroenteritis were included. However, the distribution of pathogens was not recorded in our study. Another possible reason for the longer duration of vomiting in our study is that we only included children who consulted a GP, whereas other studies also included children who did not consult a physician. A multicenter study that included 12 European hospitals previously demonstrated that vomiting was present in 20% of children on day 2 and in < 10% on day 5 [18]. These secondary care data are comparable with ours for primary care.

The studies by Roslund et al. and Reeves et al. showed that diarrheal episodes persisted for 5 to 7 days after discharge from emergency departments [19, 20]. This is consistent with the results of our study in primary care, although we add to this by providing insight into the roles of age and vomiting as a risk factor. Of note, 90% of children were free of diarrhea on day 5 in the group aged > 12 months compared to day 7 in the group aged 6–12 months. We also found no association between ondansetron use and the increase in episodes of diarrhea, as the circulating concentration of ondansetron is expected to reach 50% of its maximum serum level at 3 h after oral dosing [21].

At presentation, ≤ 40% of children had a fever, consistent with the expected course of rotavirus gastroenteritis in which low-grade fever is typically seen in 30%–50% of children [16]. Also supporting existing literature on the uncomplicated course of childhood fever in primary care, fever resolved after 4 days in 90% of children [22].

Most of the children who deteriorated did so on days 2 and 3 after presentation, in line with the findings of Friesema et al. who reported a median of 3 days to hospitalization [23]. In our study, children who deteriorated during follow-up showed higher frequencies of vomiting at presentation and during follow-up than children who recovered (9 vs 5 episodes in 24 h). In a study by Stephen et al., children with gastroenteritis referred to pediatric emergency departments also had 9 episodes of vomiting in the preceding 24 h [14]. This indicates that the frequency of vomiting is especially predictive of referral to the emergency department. Indeed, vomiting is one of the most important symptoms for considering failure of oral rehydration therapy [24]. GPs should therefore take particular care to note the frequency of vomiting at each assessment of a child with acute gastroenteritis.

## Conclusions and implications for clinicians and policymakers

To provide good safety netting advice, it is necessary that we provide a full description of the expected duration of symptoms in an average uncomplicated course of acute gastroenteritis, detailing the predictors of deterioration whenever possible. Based on the present study, we recommend that GPs at the OOH-PC educate parents about the duration of symptoms and what alarm symptoms to monitor as part of this safety netting advice. It seems reasonable to advise that vomiting should resolve within 5 days and that fever should resolve within 4 days of presentation in 90% of children. Regarding diarrhea, however, it is important to differentiate advice by the age of the child: children aged ≤ 12 months may have diarrhea for a further 7 days, but children aged > 12 months should recover within 5 days. GPs may need to monitor closely those children who have higher frequencies of vomiting at presentation because these children deteriorated more often in our study. Although further research is needed to confirm these results, the advice is consistent with good practice and the results of other research in this field.

## Supplementary Information

**Additional file 1. **Estimated percentages and confidence intervals for symptoms by age and severity of vomiting.

**Additional file 2. **Day of deterioration requiring hospital referral or admission.

**Additional file 3. **Symptoms present by day of follow-up in children who deteriorated. Results are shown as n (%).

## Data Availability

De-identified individual participant data (including data dictionaries) will be made available, in addition to study protocols, the statistical analysis plan, and the informed consent form. The data will be made available upon requests by researchers who provide a methodologically sound proposal for use in achieving the goals of an approved proposal. Proposals should be submitted to g.a.holtman@umcg.nl.

## Abbreviations

GLMM: Generalized linear mixed model
GP: General Practitioners
IQR: Interquartile ranges
OOH-PC: Out-of-hours primary care
RCT: Randomized controlled trial
