# Supplementary material for: Course of uncomplicated acute gastroenteritis in children presenting to out-of-hours primary care
Source: BMC Prim Care. 2022 May 24;23:125. doi: 10.1186/s12875-022-01739-2 (PMC9128130; doi:10.1186/s12875-022-01739-2)
Supplement: Supplementary file 1 — Additional file 1. Estimated percentages and confidence intervals for symptoms by age and severity of vomiting. [file 12875_2022_1739_MOESM1_ESM.pdf]

Additional file 1. Estimated percentages and confidence intervals for symptoms by age and severity of vomiting

(a) Vomiting

Day	Age and severe vomiting*	Estimated percentages	95% CIs	
			Lower	Upper
Baseline	6–12 months; no severe vomiting	83.2	65.9	92.6
	6–12 months; severe vomiting	100.0	–	–
	> 12 months; no severe vomiting	88.1	78.7	93.7
	> 12 months; severe vomiting	100.0	–	–
2	6–12 months; no severe vomiting	20.1	6.1	49.4
	6–12 months; severe vomiting	51.9	35.5	68.0
	> 12 months; no severe vomiting	29.1	15.4	48.0
	> 12 months; severe vomiting	37.1	27.5	47.8
3	6–12 months; no severe vomiting	26.3	9.5	55.0
	6–12 months; severe vomiting	26.5	15.2	42.0
	> 12 months; no severe vomiting	29.1	15.5	47.8
	> 12 months; severe vomiting	27.4	19.3	37.2
4	6–12 months; no severe vomiting	8.7	1.6	35.2
	6–12 months; severe vomiting	18.0	9.4	31.8
	> 12 months; no severe vomiting	12.0	4.8	26.8
	> 12 months; severe vomiting	16.5	10.9	24.3
5	6–12 months; no severe vomiting	8.5	1.8	32.1
	6–12 months; severe vomiting	12.1	5.7	23.6
	> 12 months; no severe vomiting	9.9	3.9	23.2
	> 12 months; severe vomiting	6.4	3.7	11.0
6	6–12 months; no severe vomiting	8.5	1.6	34.3
	6–12 months; severe vomiting	2.1	0.6	6.9
	> 12 months; no severe vomiting	8.2	2.9	21.3
	> 12 months; severe vomiting	2.8	1.4	5.7
7	6–12 months; no severe vomiting	0.0	–	–
	6–12 months; severe vomiting	3.0	0.9	9.4
	> 12 months; no severe vomiting	1.3	0.2	7.8
	> 12 months; severe vomiting	1.3	0.5	3.2

(b) Diarrhea

Day	Age and severe vomiting*	Estimated percentages	95% CIs	
			Lower	Upper
Baseline	6–12 months; no severe vomiting	84.7	66.7	93.9
	6–12 months; severe vomiting	49.5	36.0	63.0
	> 12 months; no severe vomiting	62.4	47.7	75.1
	> 12 months; severe vomiting	38.9	31.0	47.5
2	6–12 months; no severe vomiting	61.5	34.5	82.9
	6–12 months; severe vomiting	33.9	20.2	51.1
	> 12 months; no severe vomiting	20.1	9.5	37.6
	> 12 months; severe vomiting	18.2	11.6	27.3
3	6–12 months; no severe vomiting	65.7	35.1	87.1
	6–12 months; severe vomiting	33.2	19.4	50.7
	> 12 months; no severe vomiting	22.6	10.7	41.5
	> 12 months; severe vomiting	24.2	16.3	34.2
4	6–12 months; no severe vomiting	30.8	11.5	60.5
	6–12 months; severe vomiting	40.5	25.4	57.7

	> 12 months; no severe vomiting	6.2	1.9	18.6
	> 12 months; severe vomiting	20.6	13.4	30.4
5	6–12 months; no severe vomiting	23.7	7.4	54.6
	6–12 months; severe vomiting	21.0	11.2	35.8
	> 12 months; no severe vomiting	10.7	4.2	24.8
	> 12 months; severe vomiting	8.0	4.3	14.4
6	6–12 months; no severe vomiting	4.9	0.6	29.3
	6–12 months; severe vomiting	21.9	11.0	38.9
	> 12 months; no severe vomiting	2.1	0.3	15.3
	> 12 months; severe vomiting	9.3	5.1	16.5
7	6–12 months; no severe vomiting	10.5	2.0	40.0
	6–12 months; severe vomiting	11.2	4.8	23.9
	> 12 months; no severe vomiting	8.8	3.2	22.2
	> 12 months; severe vomiting	2.6	0.9	7.2

(c) Fever (≥38°C)

Day	Age and severe vomiting*	Estimated percentages	95% CIs	
			Lower	Upper
Baseline	6–12 months; no severe vomiting	19.5	7.6	41.6
	6–12 months; severe vomiting	21.9	12.6	35.5
	> 12 months; no severe vomiting	35.3	21.9	51.4
	> 12 months; severe vomiting	19.5	13.8	26.9
2	6–12 months; no severe vomiting	20.9	6.4	50.6
	6–12 months; severe vomiting	21.7	11.0	38.4
	> 12 months; no severe vomiting	24.5	11.9	43.9
	> 12 months; severe vomiting	28.6	19.9	39.2
3	6–12 months; no severe vomiting	0.0	–	–
	6–12 months; severe vomiting	15.5	6.8	31.3
	> 12 months; no severe vomiting	8.6	2.8	23.8
	> 12 months; severe vomiting	14.7	9.2	22.6
4	6–12 months; no severe vomiting	0.0	–	–
	6–12 months; severe vomiting	7.1	2.5	18.6
	> 12 months; no severe vomiting	2.1	0.2	15.6
	> 12 months; severe vomiting	10.5	6.1	17.4
5	6–12 months; no severe vomiting	0.0	–	–
	6–12 months; severe vomiting	3.7	1.0	12.4
	> 12 months; no severe vomiting	2.0	0.3	12.8
	> 12 months; severe vomiting	2.5	1.0	6.3
6	6–12 months; no severe vomiting	0.0	–	–
	6–12 months; severe vomiting	1.0	0.2	5.7
	> 12 months; no severe vomiting	2.1	0.3	13.2
	> 12 months; severe vomiting	2.0	0.7	5.5
7	6–12 months; no severe vomiting	0.0	–	–
	6–12 months; severe vomiting	2.3	0.5	9.2
	> 12 months; no severe vomiting	6.6	1.9	20.8
	> 12 months; severe vomiting	1.4	0.4	4.7

*Persistent vomiting was based on; 1) at least four episodes of vomiting 24 hours before presenting to the OOH-PC centre; and 2) at least one episode of vomiting in the 24 hours before presenting to the OOH-PC centre
All the measurements were corrected for medication
Abbreviations: 95% CI, 95% Confidence Interval; OOH-PC, out-of-hours primary care.
