# Supplementary figures and images for: Course of uncomplicated acute gastroenteritis in children presenting to out-of-hours primary care
Source: BMC Prim Care. 2022 May 24;23:125. doi: 10.1186/s12875-022-01739-2 (PMC9128130; doi:10.1186/s12875-022-01739-2)

**Additional file 2.** Day of deterioration requiring hospital referral or admission

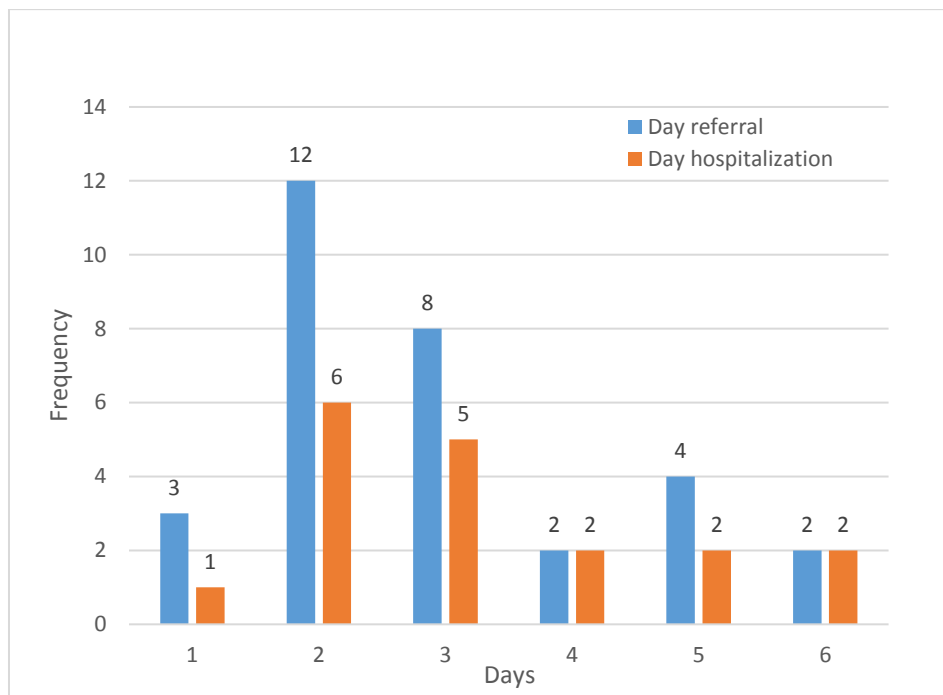

Supplement: Supplementary file 2 — Additional file 2. Day of deterioration requiring hospital referral or admission. [file 12875_2022_1739_MOESM2_ESM.pdf]
