# Supplementary material for: Course of uncomplicated acute gastroenteritis in children presenting to out-of-hours primary care
Source: BMC Prim Care. 2022 May 24;23:125. doi: 10.1186/s12875-022-01739-2 (PMC9128130; doi:10.1186/s12875-022-01739-2)
Supplement: Supplementary file 3 — Additional file 3. Symptoms present by day of follow-up in children who deteriorated. Results are shown as n (%). [file 12875_2022_1739_MOESM3_ESM.pdf]

Additional file 3. Symptoms present by day of follow-up in children who deteriorated

	Baseline	2	3	4	5	6	7
**Deterioration follow-up (n = 31)**							
Vomiting	29 (93.5)	18 (58.1)	19 (61.3)	14 (45.2)	7 (22.6)	3 (9.7)	1 (3.2)
Diarrhea	14 (45.2)	11 (35.5)	12 (38.7)	13 (41.9)	8 (25.8)	5 (16.1)	0 (0.0)
Fever	11 (35.5)	14 (45.2)	11 (35.5)	9 (29.0)	3 (9.7)	3 (9.7)	2 (6.5)
**Hospitalized follow-up (n = 18)**							
Vomiting	17 (94.4)	9 (50.0)	12 (66.7)	11 (61.1)	3 (16.7)	1 (5.6)	1 (5.6)
Diarrhea	10 (55.6)	4 (22.2)	9 (50.0)	8 (44.4)	5 (27.8)	2 (11.1)	0 (0.0)
Fever	4 (22.2)	8 (44.4)	5 (27.8)	5 (27.8)	2 (11.1)	2 (11.1)	1 (5.6)

Results are shown as n (%)
